# The Prevention of Tuberculosis1Read before the Medical Society of the County of Erie, June 11, 1895.

**Published:** 1901-07

**Authors:** B. G. Long

**Affiliations:** Buffalo, N.Y.; President of Buffalo Society for Prevention of Consumption


### THE PREVENTION OF TUBERCULOSIS.[1]

By B. G. LONG, M. D., Buffalo, N. Y.,
President of Buffalo Society for Prevention of Consumption.

WHEN we appreciate its prevalence and frightful mortality, no argument is necessary to show that the prevention and extermination of consumption is one of the most weighty questions which confronts the medical profession today.

In our city, in the year 1894, there were 605 deaths from tuberculosis reported, including those reported as chronic bronchitis, congestion of lungs and hemoptosis, out of a total mortality of 5,280, or about 11 per cent. In 1893 the deaths from the same cause were 632, out of a total of 5,711, or again about 11 per cent. If we

1. Read before the Medical Society of the County of Erie, June 11, 1895.

should have all cases otherwise named, which are in fact tubercu-lous, included the rate would be very materially increased.

In the same years, 1894 and 1893, there were 203 deaths from diphtheria, including croup, in the former and 223 in the latter, or about one-third of the number due to tubercular diseases. We thus see that the mortality is three times greater from tuberculosis than from diphtheria, which has received so much attention from the profession as well as from health authorities and legislators with telling effect. In order to ascertain the prevalence of consumption, we will assume that the report of the secretary of the State Board of Health, that its average duration is two years, is correct. On that basis, if we add together the deaths from consumption for 1894 and 1893, which is 1,237, we will have the average number of cases of the disease at any one time.

In the month of April, 1895, there were only 38 cases of diphtheria, including croup, reported. If we allow one month as the average duration of this disease, we had approximately for that month 38 cases of diphtheria to something over 1,200 cases of consumption.

That consumption is contagious through the medium of the tubercle bacilli, found principally in the sputum, is now generally accepted by the leading men of the profession, although the general public are still densely ignorant on this question. For hundreds of years some physicians have believed that consumption was contagious, basing their belief on the instances where a husband or wife, previously healthy contracted the disease in some way from the other who was consumptive, and also where healthy nurses contracted the disease after closely attending a consumptive for a long time. We have, also, the history of legislation against its spread, over 100 years old.

In 1782 the sovereign of the Kingdom of Naples issued a decree relating to tuberculosis, containing seven propositions, in substance as follows: (1) When the physician had established that there was ulceration of the lungs in any case, he was to report the patient under penalty of 300 ducats for the first offense, and banishment for ten years for the second offense. (2) The authorities were to make an inventory of the clothing found in the patient's room, which was to be identified after his death. If any opposition was made, the person offering it was to have three years in the galleys or in prison, if he belonged to the lower class, three years in the castle and 300 ducats if he belonged to the nobility. (3) Household goods which are not susceptible to contagion were to be immediately cleansed, while those susceptible were to be at once burned. (4) The authorities themselves were to tear out and replaster the house, alter it from cellar to garret, carry away and burn the doors and wooden windows and put in new ones. (5) The sick poor were to be at once removed to a hospital. (6) Newly built houses could not be inhabited for one year from their completion, and for six months after plastering had been finished and repairing done. (7) Superintendents of hospitals must keep in separate places clothing and bedding for the use of consumptives.

Other severe penalties were threatened to those who bought or sold articles which had been used by consumptives, and to any transgressor whomsoever. The results following this decree were wonderful. It is estimated that in 1782 the mortality from tuberculosis in the Kingdom of Naples was 10 to each 1,000 of population. In 1887 the official statistics for the Neapolitan kingdom showed that the death-rate from all forms of tuberculosis was only 2.05 per 1,000.

The germ theory being unknown at that time, and other means of diagnosis being crude, an early recognition of the disease was unusual, and consumptives were not pronounced such until well along in the disease, as is still often the case with practitioners who are either unable to make an early diagnosis or hesitate to alarm the patient or his friends until forced to do so by the gravity of the case.

It remained for Koch in 1882, however, to establish the fact that the tubercle bacilli is the active agent in the spread of this scourge, and also make it possible to diagnosticate pulmonary tuberculosis in almost every instance in its incipiency.

In our study of this subject we are not only to consider means to prevent the spread of the disease from the infected to the healthy, but also means to check the progress of the disease in the infected individual.

Dr. Nuttall, of Johns Hopkins Hospital, counted 4,312,581,280 bacilli in the expectoration of a consumptive in twenty-four hours. Heller claimed that a person with consumption would expectorate 7,200,000,000 of bacilli in a day, and that in a dried state they would live over six months and in a moist, putrefying substance 43 days. This may be overdrawn, but whatever the number and their longevity, the fact remains that with 1,200 cases in our city, practically unrestricted in any way, there are enough living bacilli floating about in the air to infect every susceptible citizen as well as the stranger within our gates.

When we consider the fact that cases of that dreaded disease, diphtheria, are ordinarily of short duration and that the patients are confined to the house and usually to their beds, and the danger of contagion being common knowledge, patients were in a large measure isolated even before the important placarding of houses was inaugurated; and, on the other hand, we know that tubercular diseases vastly more numerous are, as a rule, slow in their progress, and that from the first, long before the victim gives up his business or social engagements, he is measurably a source of contamination and a menace to all with whom he associates, and when we realise how ignorant the public and even many physicians are of the danger of contagion, can we appreciate in some degree the importance of this subject.

What has our State Board of Health done towards the extermination of this disease? They make the boast that we are the first state to inaugurate measures to exterminate the disease from our herds, and have secured appropriations for this work and have carried it on to such an extent that a 500-page book is required to give the report upon that work for 1894. The same report gives the number of deaths from consumption reported as such in the year 1894, as 13,123, surely far short of the actual deaths from tuberculosis, and yet I am unable to learn that one word has been uttered by this board warning the public of the danger of its spread amongst human beings.

Thus far the State of Michigan, is the only state to adopt measures against the spread of this disease, excepting from animals. Its State Board of Health adopted the following resolution September 30, 1893:

*Resolved*, That hereafter consumption—and other diseases due to the bacillus tuberculosis—shall be included in the official list of "Diseases Dangerous to the Public Health," referred to in sections 1675 and 1676, Howell's statutes, requiring notice by householders and physicians to the local health officer, as soon as such disease is recognised.

And in these cases the local boards and health officers were to use their own judgment as regards the isolation and control of each case. The state board has also petitioned the legislature for a state hospital for consumptives, and is freely circulating printed information and instructions against its spread.

The health departments of many of our cities are alive to the importance of taking decisive action toward exterminating this disease. In New York City circulars and bulletins are circulated in different languages to inform the public of its dangers, and also calling attention to the curability of the disease, if properly treated in its incipiency. The health department is also equipped to disinfect contaminated articles free of expense, and will send inspectors to instruct the family, where the disease exists, in methods to prevent its spread, if so requested by the attending physician. The department is also prepared to examine sputum for tubercle bacilli upon the presentation of specimens.

In Philadelphia the health department is prepared to disinfect contaminated articles at a small fee and is disseminating literature. In the latter city, also, under the inspiration and presidency of Dr. Lawrence F. Flick, a society for the prevention of consumption has been organised, which has in its membership not only physicians, but any person who pays $1.00 or more into its treasury shall be a member for the year thus paid for. Its objects are largely educational, both to the affected individual and to the public, so that the health department may be supported in its work and proper laws enacted and enforced. It also furnishes hospital treatment to poor consumptives so far as it is able.

Dr. Flick recommends the formation of similar varieties in other cities, and I believe the suggestion a good one. I am of the opinion that such a society in our city would soon become popular, and would accomplish very much in the way of properly molding public opinion in its attitude to this question.

Through the efforts of some of the members of this society, Erie County is now erecting a separate building for the isolation and treatment of consumptives, which is a step in the right direction. Already our health department is prepared to examine sputum, and I am informed that they are continually called upon to do it. Preparations are also being made to institute active measures against the spread of this disease, by this department.

As an early diagnosis is of the utmost importance in the control and treatment of consumption, an ordinance, or still better if possible, a state law, should be enacted, making the reporting of even suspected cases of this disease mandatory, and placing them under the inspection and supervision of the health authorities. The sputum of the suspected individual, and also of every member of the household should be examined by the department bacteriologist. Cases that are well advanced in the disease should be sent to special hospitals unless they can be properly cared for at home without risk to others. Incipient cases, where the patient is still able to work, or care for himself, should, if possible, be induced to go to a more suitable climate, where his chances for recovery would be greatly increased.

Our aim should be, especially among the poor, and it is here that we find the disease most prevalent, to have them go to a suitable climate early, when they are in comparative health and are able to earn their livelihood and care for themselves. After a patient is bed-ridden or nearly so and requires a nurse or attendant, traveling is out of the question, for those who have little or no means, and it is to this class especially that I wish now to call your attention.

The greatest obstacle today, in my judgment, in the control and treatment of this disease intelligently, and in accordance with the recent advances in its study, is our inability to send those without means away to a more congenial climate.

Recent investigations by foreign physicians who sent commissioners to various countries of the globe, showed the Rio Grande region of New Mexico to be the section presenting in the highest degree the necessary conditions for combating consumption and allied diseases. Here it is that we find nearly continuous sunshine and a dry atmosphere. Mr. W. S. Burke, the United States weather observer at Albuquerque, N. M., states that not above two days in three years occur, in which the sun is not observed for the entire day at that station. The rainfall not only in New Mexico, but all of the Rocky Mountain region is very light. Undoubtedly many patients have been sent to Colorado and other resorts in that region by members of this society, who have been greatly benefited thereby. I have had two cases within six months that have stimulated me to a study of this subject. Both were given careful physical examinations by members of this society of recognised ability, who reported in both instances their inability to detect any signs of lung trouble. I found the bacilli numerous in both cases upon examination of the sputum, and had my findings verified by the city bacteriologist. One patient had means and was sent to Colorado, where he is doing well. The other one was financially unable to go away and has been obliged to remain here and work for a living. The latter is improved in health and may recover, but I think the chances for recovery are very much less for the one remaining here than for the one now in Colorado.

Throughout the Rocky Mountain region, and also in our Adirondacks, there are sanitariums where people of means can be well cared for; but what are we to do with our poor patients? To send our patients who have no means out west among strangers, where at the present time employment is almost out of the question, on account of the stringent times, which are felt more there than here, and have them suffer more from lack of food and shelter than they gain by the change of climate, so that finally they would become public charges upon an already overburdened community, ought not to be thought of.

Before sending such patients away, work must be provided for them whereby they may be self-sustaining. If work can be guaranteed them with the chances of regaining their former health immeasurably increased, we will have little trouble to get them to carry out our wishes.

I am satisfied that the providing of such work is not an unsurmountable difficulty, for fortunately since the system of irrigation has been so successfully adopted in that section, immense tracts of land have been brought under cultivation, and with such absolute control of the water supply the land produces two or three crops each season, and it therefore requires a comparatively small plot for an individual to work in order to maintain himself.

In a recent conversation with a gentleman who is largely interested in irrigation in Southern Colorado, where their company have 5,500 acres under irrigation, I am informed that such land can be bought for $40 per acre, with perpetual water rights. Land could in all probability be leased for a term of years at a very reasonable figure, with the privilege of purchasing at any time.

Our state is providing a colony for epileptics and is constantly caring for the insane. Why should it not establish colonies for consumptives in various desirable health resorts? Why is it not perfectly feasible to establish such colonies in suitable localities where under stringent medical supervision those of small means might be sent while still able to work and where, in outdoor occupation, in the pure air and sunshine, they might earn a living, and thus freed from worry and homesickness, regain their health. Such a colony ought to become self-sustaining in a very short time, for no one should be sent there who is unable to work.

In England, the Salvation Army has engaged in a somewhat similar undertaking at the mouth of the Thames, to provide homes and employment for the poor of London, but on a soil which is much less productive. I am told by an officer of the army that it has passed the experimental stage and, at the present rate of productiveness, will be self-sustaining in a very few years.

A small colony might be started in an experimental way by a society, or city or state, under proper medical supervision and control, so that all sputum and other contagious excreta would be destroyed. This colony could be added to from time to time by additional land or the establishment of industrial enterprises for the employment of the various trades, and others could be established in higher and lower attitudes in that region to suit the requirements of individual cases.

I have purposely avoided any reference to treatment by drugs, and the detail of restrictive measures, but have tried to show the reasonableness of the following conclusions:

1. The contagiousness of consumption being practically admitted, its prevalence and terrible mortality make the study of its prevention the most important question before the medical profession to-day.

2. That legal enactment should require the reporting of all cases to the health authorities, who should be authorised to inspect such cases and supervise their control, and also compel all members of an infected household to have their sputum examined by the city bacteriologist.

3. That societies similar to the one now existing in Philadelphia for the prevention of consumption should be formed to interest the public in and educate it upon this important question, and to coöperate in every way possible with the health authorities in the discharge of their duties.

4. That all cases that are well advanced should be placed in suitable hospitals, if not so situated that they can be cared for at home without the risk of infecting others.

5. That all patients with consumption detected in its incipiency should be urged to go to a favorable climate, and that colonies should be established—ultimately by the state—where those who have no means could be employed at out-door work to maintain themselves, such colonies to be under rigid medical supervision.

Note.—On March 23, 1901, a sanitarium along lines suggested in my paper, "The Rocky Mountain Industrial Sanitorium," was incorporated at Denver, Colorado, with Hon. Wm. H. Gabbert, Associate Justice Supreme Court of Colorado; Hon. Charles Hartzell and A. M. Holmes, M.D., as incorporators.

It is intended that people of little means shall be received and cared for at cost, with an opportunity for them to engage in such light employment as they are able to perform to assist them in their maintenance.

Strict medical supervision is to be enforced so that all sanitary matters are perfect in every detail. Medical men should investigate this institution when considering the disposition of incipient cases of tuberculosis. The prospectus of the institution is now ready for distribution.

520 ELMWOOD AVENUE.

